# Validation of long-term handling and storage conditions for hematopoietic stem cell products for autologous transplants

**DOI:** 10.25122/jml-2022-0230

**Published:** 2023-04

**Authors:** Anfal Mohanna, Amani Owaidah, Ahmed Albahrani, Sara Aldossary, Norah Almulhem, Hani Almohanna

**Affiliations:** 1 Department of Pathology and Laboratory Medicine, King Fahad Specialist Hospital, Dammam, Saudi Arabia; 2 Department of Clinical Laboratory Sciences, College of Applied Medical Sciences, Imam Abdulrahman bin Faisal University, Dammam, Saudi Arabia; 3 Research Center, King Fahad Specialist Hospital, Dammam, Saudi Arabia

**Keywords:** hypothermic storage, stem cells, HPSCs, cryopreservation, hematopoietic stem cells, validation

## Abstract

Hematopoietic stem cells (HPSCs) are multipotent stem cells that can differentiate into lymphoid and myeloid progenitors, giving rise to white blood cells (WBCs), red blood cells (RBCs), and platelets. HPSCs are a widely used treatment for many hematological non-malignant and malignant disorders. HPSCs can be used in the fresh or cryopreserved state for future use. Fresh HPSCs are typically stored at 2–6°C for up to 72 hours and are primarily used for allogeneic transplants or autologous transplants in myeloma and lymphoma patients. However, in some cases of autologous donations, HPSC transplantation is delayed more than three days after collection. In such situations, the cells are thawed after short-term preservation, resulting in a 35% cell viability loss. This study aimed to investigate the quality of HPSCs products after long-term storage exceeding 72 hours. The quality of HPSCs products was assessed by measuring viable CD34+ cell count, the total number of nucleated cells (TNC), and HPSCs recovery after different storage intervals of up to 120 hours in hypothermal storage. The mean total cell viability decreased by 2.18% within 72 hours and 7.4% within 120 hours, while mean CD34+ cell recovery was 92.61 % at 72 hours and 83.83 % at 120 hours in hypothermal storage. The mean TNC recovery was 89.93% at 72 hours and 76.18 % at 120 hours. All products were free from bacterial contamination for up to 120 hours under hypothermal storage conditions.

## INTRODUCTION

Hematopoietic stem cells (HPSCs) are multipotent stem cells that can differentiate into lymphoid and myeloid progenitors, giving rise to white blood cells (WBCs), red blood cells (RBCs), and platelets [1]. HPSCs are considered fresh if the product is stored at 2-6°C (hypothermal storage) without cryopreservation and utilized almost momentarily. This practice is mainly used for allogeneic transplant cases and some autologous transplants, such as myeloma and lymphoma cases [2,3]. HPSC products not scheduled for transplantation within 72 hours after harvesting must be cryopreserved. A cryoprotectant reagent such as dimethyl sulfoxide (DMSO) is typically added to preserve the product. DMSO is the most common cryoprotectant used to prevent the formation of ice crystals which can penetrate the cell membrane and induce cellular shock [4,5]. Cells are then stored in liquid nitrogen freezers in vapor or liquid phase at -140°C to -195°C for up to 10 years [6]. In 2018, a systemic review was conducted and included 109 studies that described the negative effects of DMSO in humans. It was found that DMSO may cause an adverse reaction in some patients, ranging from mild to more serious and severe events. The study also found that the DMSO percentage plays an important role in the occurrence of adverse reactions [7].

Autologous peripheral blood hematopoietic stem cells (PBHSCs) transplantation is routinely used as part of treatments to reduce the tumor burden of different malignant blood diseases such as myeloma and lymphoma [8]. Hematopoietic progenitor stem cells for clinical applications can be obtained from various sources, including bone marrow, peripheral blood, and umbilical cord blood [9]. PBHSCs are the preferred collection method due to their less invasive nature compared to other stem cell sources, such as bone marrow [10].

To mobilize hematopoietic stem cells from the bone marrow to the bloodstream, the donor is given granulocyte colony-stimulating factor (GCSF) alone or in combination with other agents for five consecutive days, after which the CD34+ cells are quantified before collection to ensure a sufficient number of CD34+ doses [11]. There are specific requirements concerning the handling and storage of non-frozen fresh stem cells, including sterility, leak proofness, and temperature stabilization at 2-6 °C [12]. In some cases where autologous lymphoma and myeloma patients require PBHSCs transplantation after 72 hours of collection, such as on day 5 following collection, the PBHSCs products are thawed after a short cryopreservation cycle. This process results in a 35% loss of stem cells [13].

To our knowledge, the total cell viability, absolute CD34+ cell count, TNC count, and recovery following the extended storage of PBHSCs after collection have not been studied. To address this gap, the key parameters and their impact on the outcomes of autologous transplants following an extended period of storage were investigated. The findings of this study could inform the standards and regulations governing the handling and storage of PBHSCs products, potentially opening up new avenues for extending beyond existing practices. This, in turn, could provide clinical benefits such as avoiding the use of cryoprotectants and reducing processing costs.

## MATERIAL AND METHODS

### Study protocol

Between July 11, 2021, and February 12, 2022, the bone marrow and stem cell transplant center at King Fahad Specialist Hospital (KFSH-D) collected 12 autologous mobilized PBHSCs using an apheresis instrument (Spectra Optia^®^ SPO, Software 11.3, Platform MNC Terumo BCT, Lakewood, CO, USA) according to established procedures [14]. All participants provided written informed consent to participate in this study. The study design protocol was approved by the Institutional Review Boards of Imam Abdulrahman Bin Faisal University (IRB-PGS-2021-01-466) and the King Fahad Specialist Hospital (IRB-LAB0319). This study was conducted under the principles of the Declaration of Helsinki.

### Product mobilization and collection

All patients were mobilized using a combination of chemotherapy (cyclophosphamide 2–4 g/m^2^ or disease-specific salvage regimens) and granulocyte colony-stimulating factor (G-CSF) (filgrastim 10 µg /kg/day). Leukapheresis was performed the day after the total blood CD34+ cell count exceeded 20 cells/µl to collect stem cells only, and no autologous plasma was collected. The pre-collection complete blood count (CBC) and whole blood CD34+ enumeration were used to determine the target collection cell dose and the total blood volume (TBV) needed for the collection process.

For this study, PBHSCs collections were processed for three to three and a half of the total blood volumes calculated. Each collection procedure lasted 3–5 hours with a final volume of 250–550 ml. The inlet flow rate ranged between 40-60 ml/min, and the stem cell flow rate varied between 1.4-2.6 ml/min. Products with an excess CD34+ cell dose of more than 2.5 × 10^6^/kg were included.

### Product preparation

Following PBHSC collection, samples for quality testing were obtained from the PBHSC bags as a control. Under sterile conditions and using a class II A2 biosafety cabinet, 5–15 ml of the PBHSCs product bag was transferred to a sterile transfer bag using a bag spike or a sterile connecting device. All products were stored in a continuously monitored refrigerator set at a temperature between 2–6 °C. Viability, CD34+ enumeration, and total nucleated cells (TNC) count were subsequently determined at 0, 72, and 120 hours. Product sterility was also evaluated at 0 and 120 hours.

### Product evaluation

Total viable CD34+ cells were determined using 7-amino actinomycin D (7-AAD) staining, which is more sensitive than trypan blue staining. Flow cytometric assays with 7-AAD are routinely used to quantify viable stem cells [15]. To ensure the accuracy of our results, we ran all samples in duplicate, and the differences between the two runs were always <10%. WBC counts were performed using a fully automated CBC analyzer (Alinity HQ) to determine the TNC counts. The CD34+ absolute count was determined by flow cytometry using a FACSCanto^TM^ cell analyzer through the single-platform method. Briefly, each sample was added to fluorochrome-labeled antibodies and CD34/CD45 reagents, which bind to the cell surface. The lyophilized pellets in BD True count tubes were then dissolved, discharging an identified number of fluorescent beads, and 7-AAD was added to assess cell viability. Ammonium chloride was added to lyse erythrocytes before the samples were obtained using a flow cytometer. Data acquisition was performed using a dual laser FACS Canto II flow cytometer (BD Biosciences), and the data were analyzed using FACS Diva software. Product sterility was evaluated at 0 and 120 h using the BACTEC^TM^ blood culture system.

### Recovery calculations

TNC and CD34+ recovery was calculated by dividing the post-storage cell count by the pre-storage cell count and multiplying by 100. The results were expressed as a percentage.

TNC recovery was calculated using the following formula:


$$=\frac{TNCRecoverypercentage\text{ (\%)}\to \text{72 }hoursor\text{ 120 }hourspoststorageTNCcount}{TNCcountatcollectionday}\times 100$$

CD34+ cells recovery was calculated using the following formula:


$$=\frac{CD34+Recoverypercentage\left(\%\right)\to 72hoursor120hourspoststorageCD31+cellcount}{CD34+cellcountatcollectionday}\times 100$$

### Sterility testing

To assess the sterility of the products during the extended storage, sterility testing was performed at the initial collection (0 hours) and after 120 hours of extended storage only. To evaluate the sterility of PBHSC products, 0.5 ml of each product was collected. That volume was validated at KFSH-D in May-2015 according to McFarland standard, using a wide spectrum of bacteria in different concentrations and inoculation volumes to determine the minimum volume of cellular therapy products needed for inoculating blood culture bottles without affecting the integrity of blood culture results. The results showed no growth detected in the negative culture bottles. All bottles inoculated with aerobic and anaerobic with 0.3 ml of the product turned positive within 4 days. Blood product volume had no effect on bacterial growth detection. Inoculating sterility culture bottles with 0.5 to 1 ml of cellular therapy product was sufficient to detect possible product contaminants.

The bottles were incubated in BD BACTEC™ FX blood culture system (BD biosciences, USA) for 120 hours. If no bacterial growth was detected, results were released as ”no growth detected,” which is considered sterile.

### Statistical analysis

One-way ANOVA variance was used to evaluate the statistical differences between the different storage intervals, with a significance threshold of P<0.05.

## RESULTS

### Characteristics of fresh autologous PBHSCs products

Twelve PBHSCs products were prepared in the transfer bags. All products contained a minimum of 287.9 cells/µL based on the CD34+ counts. The properties of the fresh PBHSCs products are shown in Table 1. Of the 12 products collected, 66.7% were from male autologous donors, and the remaining 33.3% were female donors.

**Table 1:** Characteristics of fresh autologous PBHSCs products (N=12).

Characteristic	Value
**Age mean±SD**	37±12.12
**Patient weight, mean±SD**	83.38±23.371
**Sex, % (n)**	M 66.7 (8)
	F 33.3 (4)
**Diagnosis, % (n)**	Myeloma 42% (5)
	Lymphoma 50% (6)
	TC 8% (1)
**Product volume mean, range (ml)**	411 (251–550)
**Processed total blood volume, range (ml)**	12595.75 (8812–19452)
**ACD-A volume mean, range (ml)**	41 (25–55)
**Hct (%)**	1.80 (0.35–5.44)
**WBC mean , range(×10^9^/L)**	98 (30.70–162)
**TNC count mean range (×10^8^)**	397.9 (162.23–855.79)
**CD34+ enumeration, mean range cells/µl**	1,522.47 (287.90–4,466)
**CD34+ dose mean, range (10^6^/Kg)**	6.78 (3.16–13.51)
**Total cells viability, % mean range**	93.45 (91.10–96)

M – male; F – female; TC – testicular cancer; ACD-A – Anticoagulant Citrate Dextrose Solution.

### Characteristics of PBHSCs following hypothermal storage for 72 hours and 120 hours

**Figure 1:**
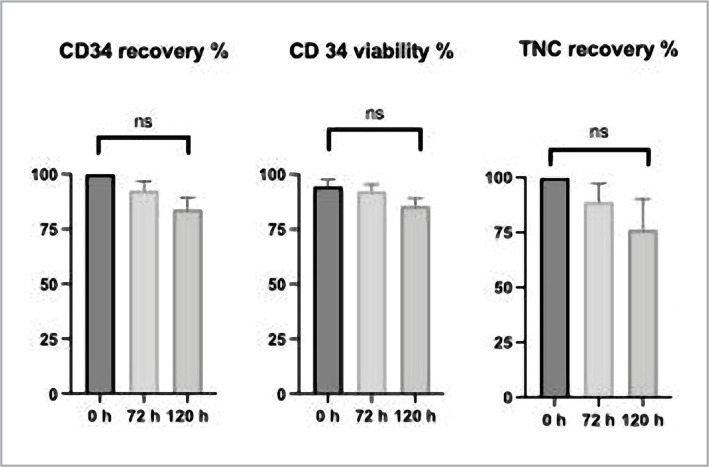
Effect of hypothermal storage (2–6°C) for up to 120 hours on PBHSC products.

During hypothermal storage at 2–6 °C, a gradual loss of total cell viability, CD34+ cell recovery, and TNC recovery were observed, but these losses were not significant. Total cell viability cells decreased by 2.18 ± 1.84% after 72 hours and by 7.40 ± 4.12% after 120 hours. The mean recovery of CD34+ reached 83.83 ± 5.35% after 120 hours. The mean TNC recovery was 89.93 ± 8.39% after 72 hours and 76.18 ± 14.09% after 120 hours, as seen in Figure 1. The stability characteristics of the PBHSC products stored for different intervals (72 hours and 120 hours) are shown in Table 2.

**Table 2:** Characteristics of hypothermal storage for PBHSC after 72 hours and 120 hours of hypothermal (2–6°C) storage (mean±standard deviation).

	72 hours of storage	120 hours of storage
**TNC Recovery, %**	89.93±8.39	76.18±14.09
**CD34+ Recovery, %**	92.61±4.17	83.83±5.35
**Total cell viability, %**	91.27±2.00	86.05±3.61

### TNC counts and CD34+ cell counts after extended hypothermal storage

No significant differences were observed between the fresh PBHSCs and those stored for 120 hours of hypothermal storage, as summarized in Table 3.

**Table 3:** Effect of hypothermal storage (2–6°C) for 120 hours on PBHSCs products.

	Collection day	After 120 hours of storage	P-value*
**WBC, ×10^9^/L**	98.53±41.16	76.00±39.96	0.188
**TNC, ×10^8^**	397.91±209.76	313±197.14	0.318
**CD34+ enumeration, cells/µl**	1,522.47±1,178.32	1,292.36±1,019.10	0.614

* – P-values reflect the difference between the fresh PBHSCs product and those stored at 2–6°C for 120 hours.

### Sterility testing

BACTEC^TM^ blood culture instrument was used to evaluate the sterility of the PBHSCs on the collection day and 120 hours after hypothermic storage. All products tested negative for bacterial contamination.

## DISCUSSION

The main objective of this study was to investigate the efficacy and quality of PBHSCs products after long-term storage beyond 72 hours without cryopreservation. Previous studies have shown that hypothermal storage (2–6°C) is more effective in maintaining cell viability and quality of PBHSCs products compared to room temperature (20–25 °C) for up to 72 hours [16,17]. The results of this study are in line with previous studies maintaining cell viability under hypothermal storage even beyond 72 hours. Providing the best storage conditions for PBHPSCs products is essential to maintain graft quality and directly impacts clinical outcomes. Successful stem cell engraftment depends on the cellularity and viability of the stem cell products [18]. However, only a limited number of studies have investigated the potential for extended storage of PBHSCs [16].

In this study, we aimed to investigate the quality and viability of non-cryopreserved PBHSCs products stored at 2–6 °C for up to 120 hours based on various quality metrics such as total cell viability, absolute CD34+ cell counts, and TNC counts. The results demonstrate the high quality of non-cryopreserved PBHSCs products stored at 2–6 °C for up to 120 hours, without significant differences compared to the fresh samples for all the studied quality and viability metrics (Fig.1). Araujo et al. assessed the quality of 11 PBHSCs products following cryopreservation and storage at 2-6 °C for up to 92 hours. While cryopreserved products had a low viable CD34+ cell recovery rate, the products stored at 2-6 °C for up to 92 hours preserved the product's quality with a CD34+ recovery of 90.1% in hypothermal storage, and the viability of the total cells was decreased only by 7% after 96 hours of hypothermal storage [16].

Several studies highlight the benefits of using fresh autologous stem cell products over cryopreserved ones for transplantation. For example, fresh products show faster engraftment with a lower incidence of neutropenia than cryopreserved products [18-20]. Furthermore, cryopreservation causes physiological changes in stem cell products, including cell aggregation [19]. In the majority of studies, the duration of fresh hypothermal storage is 48-72 hours for myeloma and lymphoma patients, indicating the potential benefits of extending the shelf life of fresh PBHSCs products [20]. This is especially true for some conditioning protocols that require additional days until transplantation. It also applies in case of unexpected delay of stem cell transplantation or poor stem cell collection that needs more than three collections. In addition to the clinical benefits, fresh stem cell products are less expensive than cryopreserved products as the thawing cycle, and the use of cryoprotectants is no longer required.

A study conducted by Derwood Pamphilon, reviewing the guidelines for high-quality storage of HPSC, recommended that processing laboratories use validated procedures that account for critical variables such as cell concentration, temperature, the interval from collection to cryopreservation, and any manipulations performed. Furthermore, they suggested assessing the product quality by measuring the CD34+ absolute cell count, HPSC cell dose, cell viability before the infusion, and evaluation of the engraftment after the infusion. They recommended cryopreserving the HPSC product if the fresh storage exceeds 48 hours [21].

Successful engraftment was observed within 28 days on four PBHSC products after 120 hours in hypothermal storage included in this study. The platelet count was 102.33 x10^9^ /L ± 49.90 x10^9^ /L, and the absolute neutrophil count was 1.34 x10^9^ /L ± 0.48 x10^9^ /L. Moreover, previous studies have shown that fresh autologous PBHSCs transplantations are less expensive [20]. A study conducted by Mohamed et al. compared the outcome of a fresh and cryopreserved autologous HPSCs transplantation in lymphoma patients. The platelets and neutrophils recovery was faster for those who received the cryopreserved graft. However, the two groups showed successful engraftment by day twenty post-transplantation. Mortality rate, the incidence of relapse, progression-free survival (PFS), and overall survival (OS) were investigated, none of which showed any differences between the two groups [22].

Consistent with previous studies, our data showed positive outcomes in prolonging the shelf life of fresh PBHSC products, especially for those conditions that required extended storage prior to transplantation.

The most reliable indicator of a transplant's success is the CD34+ cell count [18]. It is best to carry out PBHSCs transplantation as soon as possible following collection. Despite this, our findings demonstrate a good CD34+ cell recovery rate of 83.83 ± 5.35% following a 120-hour of hypothermal storage.

## Conclusion

Extended hypothermal storage for up to 120 hours has little to no impact on the quality of PBHSCs. Future research should focus on investigating the extended storage of hematopoietic stem cells from other sources, such as bone marrow and cord blood, and use a larger sample size, given that cellular components may vary from different sources. Quality assessment should also include TNC counts and sterility testing, in addition to CD34+ count and viability. The inclusion of in-vitro assays as part of functionality testing could further enhance the quality assessment of stored PBHSCs products.

## Data Availability

Further data is available from the corresponding author on reasonable request.
